# A multicentric, single arm, open-label, phase I/II study evaluating PSMA targeted radionuclide therapy in adult patients with metastatic clear cell renal cancer (PRadR)

**DOI:** 10.1186/s12885-023-11702-8

**Published:** 2024-02-01

**Authors:** David Kryza, Armelle Vinceneux, Anne-Sophie Bidaux, Gwenaelle Garin, Delphine Tatu, Claire Cropet, Jean-Noël Badel, David Perol, Anne-Laure Giraudet

**Affiliations:** 1 Hospices Civils de Lyon, Lyon, France; 2 UNIV Lyon-Université Claude Bernard Lyon 1, LAGEPP UMR 5007 CNRS Villeurbanne, Villeurbanne, 69100 France; 3 Department of Medical Oncology, Centre Leon Bérard, Lyon, France; 4 Department of Clinical Research, Centre Leon Berard, Lyon, France; 5 Department of Biostatistics, Centre Leon Berard, Lyon, France; 6 Lumen Nuclear Medicine Department, Centre Léon Bérard, Lyon, France; 7 Centre de médecine nucléaire Lumen, 15 rue Gabriel Sarrazin, cedex 08, Lyon, 69373 France

**Keywords:** ^177^Lu-PSMA-1, Radiolabelled therapy, mccRCC, PFS, Renal cancer

## Abstract

**Background:**

Despite advancements in managing metastatic clear cell renal carcinoma (mccRCC) through antiangiogenic tyrosine kinase inhibitors and immunotherapy, there remains a demand for novel treatments for patients experiencing progression despite the use of these medications. There is currently no established standard treatment for patients receiving third therapy line. Prostate Specific Membrane Antigen (PSMA) whose high expression has been demonstrated in metastatic aggressive prostate adenocarcinoma is also highly expressed in neovessels of various solid tumors including renal cell carcinoma (RCC): 86% of clear cell RCC, 61% of chromophobe RCC, and 28% of papillary RCC. Therefore, PSMA may be a target expressed in metastatic ccRCC for radionuclide therapy using PSMA ligands radiolabeled with Lutetium-177 (PRLT). ^177^Lu-PSMA delivers ß-particle radiation to PSMA-expressing cells and the surrounding microenvironment with demonstrated efficacy in metastatic prostate cancer.

**Methods:**

This is a multicenter phase I/II study designed to assess the tolerability and effectiveness of 177Lu-PSMA-1 in individuals with PSMA-positive metastatic clear cell renal cell carcinoma (ccRCC), identified through 68Ga-PSMA PET, conducted in France (PRadR). 48 patients will be treated with 4 cycles of 7.4 GBq of ^177^Lu-PSMA-1 every 6 weeks. The primary objective is to evaluate the safety of ^177^Lu-PSMA-1 (phase I) and the efficacy of ^177^Lu-PSMA-1 in mccRCC patients (phase II). Primary endpoints are incidence of Severe Toxicities (ST) occurring during the first cycle (i.e. 6 first weeks) and disease Control Rate after 24 weeks of treatment (DCR24w) as per RECIST V1.1. Secondary objective is to further document the clinical activity of 177Lu-PSMA-1 in mccRCC patients (duration of response (DoR), best overall response rate (BORR), progression fee survival (PFS) and overall survival (OS).

**Discussion:**

Our prospective study may lead to new potential indications for the use of ^177^Lu-PSMA-1 in mccRCC patients and should confirm the efficacy and safety of this radionuclide therapy with limited adverse events. The use of ^177^Lu-PSMA-1may lead to increase disease control, objective response rate and the quality of life in mccRCC patients.

**Trial registration:**

ClinicalTrials.gov: NCT06059014.

**Supplementary Information:**

The online version contains supplementary material available at 10.1186/s12885-023-11702-8.

## Background

Kidney cancer is a common kind of tumor in the world with an estimated number of 431, 288 new cases in the word of approximately [1]. Kidney cancers represented 2% of all cancers affecting twice as many men and women. Renal cell carcinoma is one of the most prevalent tumor types [2] accounting 60–75% of renal cell carcinomas [3]. It occurs in sporadic form in 95% of all cases and a minor part of them are associated with von Hippel-Lindau disease and other familial syndromes. Comprising papillary and chromophobe subtypes, these subtypes collectively represent over 90% of all renal cell carcinoma (RCC). Clear cell RCC (ccRCC) stands out as one of the most aggressive histologic subtypes within kidney cancer. Approximately 30% of ccRCC patients present with metastases at the time of diagnosis, and around 60% develop metastases within the initial 2–3 years after diagnosis. Metastasis constitutes a primary factor contributing to mortality in individuals with ccRCC.

The treatment approach for metastatic ccRCC revolves around antiangiogenic targeted therapies such as VEGF Receptor tyrosine kinase inhibitors (TKIs), checkpoint inhibitors, and combinations of checkpoint inhibitors with TKIs or dual checkpoint inhibitors.

Up to a couple of years ago, first line treatment was based on VEGF Receptor TKI, such as sunitinib or pazopanib with median progression free survival (mPFS) in the pivotal clinical trials ranged from 8 to 11 months and median overall survival (mOS) ranged from 25 to 29 months [4, 5]. Based on large phase III trials, first line treatment has now changed to combination treatments improving outcome for patients with advanced ccRCC. In individuals with intermediate/poor risk disease, the combination of ipilimumab and nivolumab can be used as part of the treatment approach. Median PFS in the CHECKMATE 214 was 11.2 months (IC95% 8.4–16.1) and median OS 48.1 months (IC95% 35.6-NE) in the intermediate/poor risk group [6]. The combination pembrolizumab plus axitinib is also approved for the first line treatment of RCC [7]. Other combinations of checkpoint inhibitors and anti-VEGFR TKI have been studied with promising results, such as avelumab + axitinib, pembrolizumab + lenvatinib and nivolumab + cabozantinib [8–10]. However, prospective data on 2nd and 3rd line treatments are disappointing. In the pivotal trial of cabozantinib versus everolimus, in patients treated with ≥ 1 prior VEGFR TKI, overall response rates (ORR) were 17% vs. 3% with cabozantinib and everolimus respectively. Median PFS was 7.4 months (95% CI 6.6–9.1) in the cabozantinib group and 3.9 months (3.7–5.1) in the everolimus group. Median OS was 21.4 months (95% CI 18.7–not estimable) in the cabozantinib group and 16.5 months (14.7–18.8) in the everolimus group [11]. In the CHECKMATE 025 trial comparing nivolumab to everolimus in patients treated with ≥ 1 prior VEGFR TKI, ORR was 22.9% (95% CI, 18.9-27.3%) with nivolumab and 4.1% (95% CI, 2.4-6.5%) with everolimus. Median PFS was 4.2 months (95% CI 3.7–5.4) in the nivolumab arm and 4.5 months (3.7–5.5) in the everolimus group. Median OS was 25.8 months (95% CI 22.2–29.8) in the nivolumab arm and 19.7 months (17.6–22.1) in the everolimus arm. The Data on 2nd and 3rd line therapy for patients with metastatic ccRCC treated with combinations are limited. In this context, it will be of great interest to propose new therapies to patients with metastatic clear cell renal cancer.

PSMA (Prostate-specific membrane antigen), also known as glutamate carboxypeptidase II, is a type 2 transmembrane glycoprotein that is overexpressed in prostate adenocarcinomas [12] and highly expressed in the endothelium of tumor-associated neovasculature [13]. In this context, radiolabeled PSMA-targeting was introduced to enhance nuclear medicine imaging and to be used as theranostic agents for patients with metastatic prostate cancer [13, 14]. Recently, there is a growing interest in the clinical evaluation of PSMA-PET imaging in detecting ccRCC and its metastases [15–17] since PSMA-PET imaging showed superior performance in the staging or diagnostic of recurrence in patient with RCC. Thus, the focus on targeting neovessels with 177Lu-PSMA-1 holds significant promise. The use of 177Lu-labeled PSMA ligands has shown promising and encouraging results in the context of metastatic prostate cancer [17, 18]. Considering the notable improvement in the prognosis of metastatic RCC patients over the last decade with the use of anti-angiogenic therapy, radionuclide therapy targeting neovessels in ccRCC becomes particularly intriguing. While the safety of 177Lu-PSMA treatments in metastatic prostate cancer has been well-documented, it is noteworthy that such safety data is currently lacking for renal cancer.

Given all these data, our proposal involves initiating a phase I/II study, incorporating a safety run-in phase, to assess both the tolerability and efficacy of 177Lu-PSMA-1 in individuals diagnosed with PSMA-positive metastatic ccRCC, identified through 68Ga-PSMA PET as define below. Eligible patients will be treated with a maximum of 4 cycles of 7.4 GBq of ^177^Lu-PSMA-1 every 6 weeks. We will also investigate the capacity of ^68^Ga-PSMA PET to predict ^177^Lu-PSMA-1 tumor response. Finally, exploratory study will include radiobiological biomarkers reflecting radiosensivity susceptibility (dosimetry study). Studying these biomarkers in a population of RCC patients is crucial as genes alteration may be more frequent in these groups of patients. They may predict toxicity but also tumor response in RCC.

## Methods

### Study design and objectives

This study adopts an open-label Phase I/II design following a Fleming methodology to explore the safety and efficacy of repeated cycles of 177Lu-PSMA-1 in patients with metastatic clear cell renal cell carcinoma (ccRCC). The study comprises two distinct phases:

- Safety Run-In Part: Evaluate the safety of 177Lu-PSMA-1 (Initiate with 6 patients receiving the starting dose of 4 cycles of 7.4 GBq of 177Lu-PSMA-1, administered every 6 weeks). If more than one patient encounters severe toxicity during the first cycle, a lower dose of 177Lu-PSMA-1 will be assessed in an additional cohort of 6 patients. The 6 patients in this safety run-in phase, treated at the selected dose for Phase II, will be included in the Phase II evaluation.
- A phase II part aiming to assess the clinical activity of ^177^Lu-PSMA-1.

For all parts: Patients with metastatic ccRCC will be selected, treated and followed-up as: Selection step with ^68^Ga-PSMA PET: A selection step is mandatory for all patients to evaluate the PSMA expression in tumor lesions through ^68^Ga-PSMA PET and according to local imaging review. Only patients with PSMA expressing tumors will be eligible to the treatment step according to criteria defined below. Treatment step: Eligible patients will be treated with ^177^Lu-PSMA-1 (initial activity: 7.4 GBq), intravenously (IV) for 4 administrations every 6 weeks. Assessment step: To assess tumor response according to RECIST V1.1, imaging will be performed at baseline, at W9, at W24, then every 12 weeks up to 1 year after the first administration and then every 24 weeks until progression, death, and loss to follow-up, or overall study completion, whichever is earliest. Survival status will be documented for all patients until death or overall study completion at least 12 months after the last patient.

**Fig. 1:**
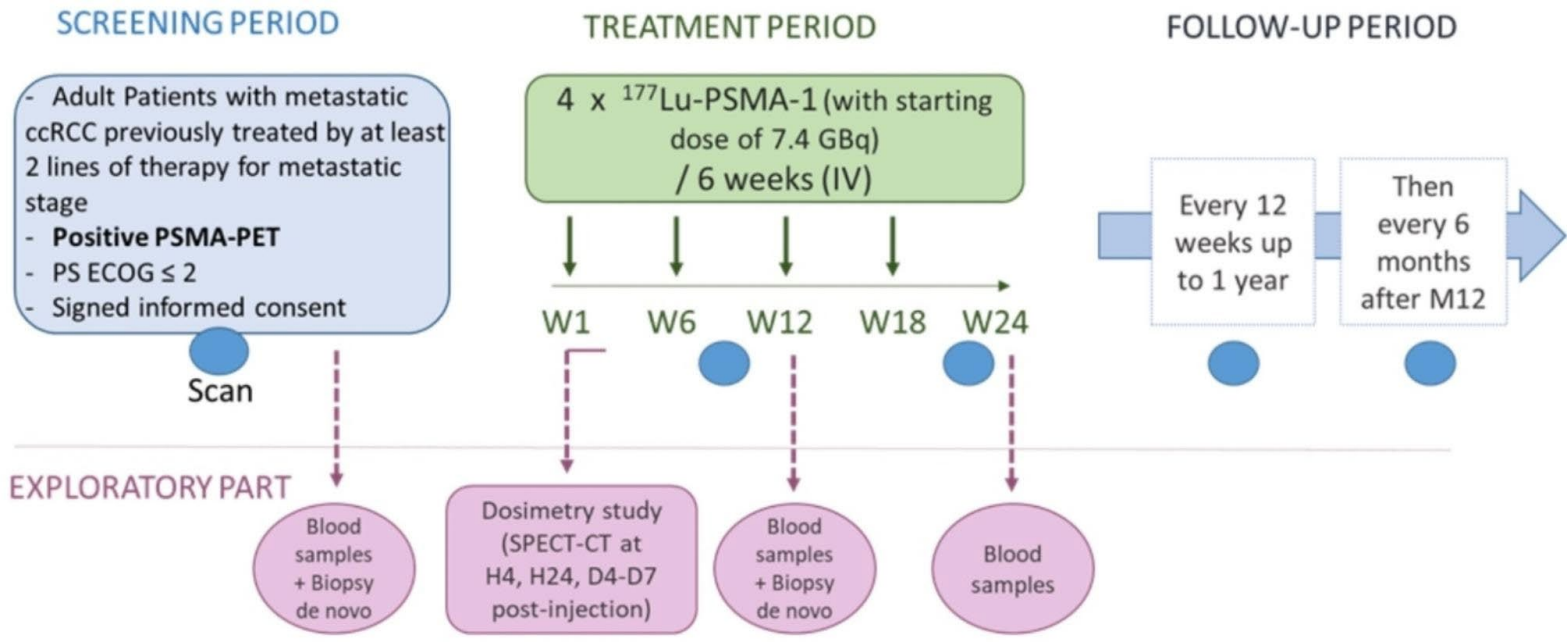
PRadR trial design

Patients with metastatic ccRCC will be selected, treated and followed-up as reported in Fig. 1.

A list of participating centers is reported in the Table 1

**Table 1:** List of participating hospitals

No	Center	Site	Principal investigator
1	Centre Léon Bérard (sponsor)	Lyon (France)	Anne Laure Giraudet (MD)
2	Centre Jean Perrin	Clermont Ferrand (France)	Hakim Mahammedi (MD)
3	CHU Bordeaux	Bordeaux (France)	Marie Meyer (MD)
4	CHU Grenoble	Grenoble (France)	Loïc Djaleb (MD)

In the safety run-in part, the primary objective will evaluate the safety of ^177^Lu-PSMA-1 in metastatic ccRCC patients. The primary endpoint will be the Incidence of Severe Toxicities (ST) defined as specific adverse events (AEs) graded using NCI CTCAE V5.0 occurring during the ST period (i.e. the 6 first weeks) and assessed as related to study drug and considered clinically significant.

In the phase II part, the primary objective will be to evaluate the efficacy of ^177^Lu-PSMA-1 in mccRCC patients. The primary endpoint will the Disease Control Rate after 24 weeks of treatment (DCR24w) defined as the rate of patients with a SD, PR or CR according to RECIST v1.1. As secondary objectives, we intend to further document the clinical activity of ^177^Lu-PSMA-1 repeated cycles in metastatic ccRCC patients, to further define the safety profile of ^177^Lu-PSMA-1 in metastatic ccRCC patients and to evaluate the benefit on health-related quality of life including pain.

Secondary efficacy endpoints are the following: Duration of Response will be measured from the time of first documented objective response (i.e., CR or PR) until the first documented progression (radiological or clinical) or death due to any cause and censored at the date of the last available tumor assessment. Progression Free Survival will be measured from the date of study drug start until the date of event defined as the first documented progression (radiological or clinical) or death due to any cause. Patients who have not progressed or died at the time of analysis will be censored at the time of the latest date of assessment from their last evaluable RECIST assessment. Overall survival will be calculated from the date of the first ^177^Lu-PSMA-1 administration until the date of death due to any cause and censored at the date of last contact for patients alive at last contact.

The assessment of safety will be based on the type, frequency, severity, and relatedness to study drug of adverse events (AEs) including ST, Serious Adverse Events (SAEs) and Suspected Unexpected Serious Adverse Reactions (SUSARs) graded using Common Terminology Criteria for Adverse Events (CTCAE) V5.0. Descriptive statistics will be provided for characterizing and assessing patient tolerance to treatment. To evaluate the benefit on quality of life and pain: Quality of life and effect on pain will be evaluated with specific questionnaires (the Functional Assessment of Cancer Therapy Kidney Symptom Index-19 (FKSI-19), the Functional Assessment of Cancer Therapy-General (FACT-G), and the EuroQol five dimensional three level (EQ-5D-3 L) instruments to be completed every 3 months for 1 year.

Management of severe or intolerable adverse events may require temporary discontinuation of therapy temporary interruption of treatment, prolongation of the interval between injections, reduction of activity (administration of reduced activity at 5900 MBq i.e. a reduction of 20% of the starting activity) or discontinuation of treatment.

The study protocol follows the SPIRIT guidelines.

### Patients

Adult patients (male or female) that are 18 years of age or older will be enrolled according to the following inclusion criteria:

- histologically confirmed diagnosis of metastatic clear cell renal cell carcinoma (mccRCC) previously treated by at least 2 lines of therapy in the advanced/metastatic setting including at least 1 line of anti-VEGFR and 1 line of immunotherapy ;
- documented radiological disease progression at the time of inclusion with measurable disease as per RECIST v1.1 ;
- Eligibility on ^68^Ga-PSMA-PET:
  - PSMA-PET positive lesions will be defined as lesions demonstrating a tracer uptake greater than physiological liver uptake on a visual analysis following Promise 2 criteria [19].
  - For patient with only extrahepatic disease: ≥ 50% of positive lesions.
  - For patient with both extra-hepatic and liver metastasis: ≥ 50% of positive extrahepatic metastatic lesions and ≥ 80% of positive supracentimetric liver metastatic lesions.
  - For patient with only liver metastatic lesions: ≥ 80% of positive supracentimetric lesions.
- Life expectancy ≥ 3 months; Eastern Cooperative Oncology Group performance status 0, 1 or 2;
- Biological criteria: Absolute Neutrophil Count (ANC) ≥ 1.5 × 10^9^/L; Platelets ≥ 100 × 10^9^/L; Hemoglobin ≥ 9 g/dL without transfusion within 7 days; Renal and hepatic function: AST and ALT ≤ 1.5x ULN (Upper Limit of Normal) in the absence of liver metastases (≤ 3x ULN if liver metastasis); Total bilirubin ≤ 1.5x ULN; Serum creatinine ≤ 1.5x ULN or creatinine clearance ≥ 40 mL/min/1.73m2 (calculated by CKD-EPI);
- Specific toxicities related to any prior anti-cancer therapy must have resolved to grade ≤ 1 (defined by the NCI CTCAE v5.0), except for alopecia and Grade 2 peripheral neuropathy or anemia (as defined in the criteria for minimal laboratory requirements);
- Information and signed informed consent prior to any protocol-specific procedures performed.
- Contraception in women and sexually active and fertile men up to 6 months after the last dose of study treatment.

Principal exclusion criteria will include:

- Patients with a history of treated brain metastases are eligible to participate, given that they demonstrate stability (no signs of progression in imaging for a minimum of four weeks before C1D1, and any neurological symptoms have reverted to baseline). Additionally, these individuals should not exhibit any evidence of new or enlarging brain metastases and are not using steroids (at doses higher than 10 mg/d of methylprednisolone or equivalent) for at least 4 weeks prior C1D1 ;
- Patient previously treated with any therapeutic radiopharmaceutical.
- Persisting toxicities related to previous anti-cancer therapy which were not resolved to grade ≤ 1 (expect: anemia provided that Hemoglobin ≥ 9 g/dL without transfusion within 7 days) and /or any persistent irAE of any grade (except adequately controlled irAE (e.g.: with replacement therapy for endocrine irAE);
- History within 2 years of cancer other than renal cancer, except for basal or squamous cell carcinoma of the skin, in-situ carcinoma of the cervix, localized prostate cancer;
- History of idiopathic pulmonary fibrosis, non-infectious pneumonitis, interstitial lung disease that required steroids or has current pneumonitis, interstitial lung disease, drug-induced pneumonitis, or evidence of active pneumonitis on screening chest CT scan;
- Administration of any anticancer treatment (immunotherapy, chemotherapy), investigational therapy within 4 weeks prior to receiving the first dose of study treatment; Surgery within 4 weeks prior to receiving the first dose of study treatment;
- Administration of Growth factors targeting the myeloid lineage (e.g. G-CSF, GM-CSF, M-CSF) or growth factors targeting the erythroid lineage (e.g. erythropoietin) within 2 weeks prior to receiving the first dose of study treatment;
- Patients with clinically significant hematuria, hematemesis, or hemoptysis exceeding 0.5 teaspoon (2.5 mL) of red blood, as well as those with a history of coagulopathy or other significant bleeding (e.g., pulmonary hemorrhage) within the 3 months prior to the initiation of the study treatment.
- Patients receiving anticoagulation medication will be eligible only if the dosage and route of administration have remained stable since at least 2 weeks prior C1D1 ;
- Patients with an active uncontrolled infection;
- Women pregnant or breastfeeding.
- Patients placed under a legal protection regimen: Judicial Safeguards, curatorship or guardianship.

### Investigational medicinal product

The synthesis of ^177^Lu-PSMA-1 will be carried out on site by the radiopharmacy of the nuclear medicine department following the manufacturing process described in the IMPD of ^177^Lu-PSMA-1 already validated by the French safety drug authority (ANSM). In brief, the synthesis of ^177^Lu-PSMA-1 consists of the metalation of the raw material [PSMA1, ITM pharmaceutical] with lutetium 177 chloride (EndolucinBeta®) in the presence of ascorbate buffer at 90 °C. The entire process is performed on an automated synthesis machine (MiniAio, Trasis®) inside a vacuum-ventilated, laminar flow, class A shielded enclosure, which allows the synthesis, dilution, sterilizing filtration and distribution of the radiopharmaceutical. ^177^Lu-PSMA-1 synthesis could be achieved in less than 40 min. ^177^Lu-PSMA-1 radiochemical purity is over 99% with a high radiochemical yield without manual processing to limit operator radiation exposure. The procedure is completely automated and can be performed several times a day.

### Sample size and statistical considerations

The PRADR study is intented to recruit up to 48 evaluable participants and is divided in two parts:

#### Safety run-in

maximum sample size of 12 patients.

Six (6) first patients will be treated at the starting dose (7.4 GBq of ^177^Lu-PSMA-1 for 4 cycles of treatment; every 6 weeks).

- If ≤ 1/6 patients experience severe toxicity (i.e. related to study drug) during the first cycle of therapy (i.e. during the first 6 weeks), the safety data will be considered acceptable and the enrolment will be continued in phase II.
- If > 1/6 patients experience severe toxicity during the first cycle of therapy, then a lower dose of ^177^Lu-PSMA will be evaluated in an additional cohort of 6 patients.

The 6 patients, enrolled in this safety run-in step and treated at the selected dose for phase II, will be included in the evaluation of Phase II part.

*Phase II*: The sample size calculation was based on a Fleming’s single stage phase II design, with a minimum success (objective response rate at 24 weeks) considered of interest of p1 = 30% and an uninteresting rate of p0 = 10%. Assuming a type I error alpha of 0.05 and 90% power, 33 patients are needed to reject the null hypothesis H0: p ≤ p0 versus the alternative hypothesis H1: p ≥ p1 in a unilateral situation.

Based on the assumption that 1)15% of screened patients may have a PSMA negative disease and 2) that among positive PSMA patients 10% may be non-evaluable for the primary endpoint, a maximum of 42 patients will be included in the study. At the time of analysis, results will be in favor of a clinical activity of ^177^Lu-PSMA-1 if the lower bound of the unilateral 95%CI of DCR24w is over 10% (this translates in at least 7 successes observed among 33 evaluable patients).

### Ancillary study

There is growing interest in dosimetry study and radiobiology biomarker analyses. To reach this goal, ancillary studies have been added to the PRADR clinical trial.

#### Dosimetry exploratory analysis

In order to follow-up the biodistribution of the ^177^Lu-PSMA-1 in patient (whole-body) and in the tumor over time with a high sensitivity, a dosimetry study will be performed to quantify the activity distribution (time-activity curve) in function of the time after injection from counts number measured using regions of interest (ROI) techniques for each organ or tumor lesions. Repeated SPECT-CT acquisitions will be performed on the first 6 patients treated at Centre Léon Bérard as follow (optional for the other CLB patients according to their consent and for patients enrolled in other participating centers):

- At cycle 1:
  - H4 after injection of ^177^Lu-PSMA-1.
  - H24 after injection of ^177^Lu-PSMA-1.
  - Between D4 and D7.
- At cycle 2, 3 and 4:
  - H4 or H24 after injection of ^177^Lu-PSMA-1 as per institutional practice.

#### Biological exploratory analysis

RCC appear to be frequently associated with a DNA damage repair capacity alteration. Patients presenting a delayed of radiation-induced nucleo-shuttling of the ATM kinase (RIANS), a major actor of the DNA double-strand break repair and signaling have been shown to be more radiosensitive through a very robust and documented predictive assay [20, 21] whose clinical applicability has been demonstrated [22]. The advantage of this approach is to evaluate both healthy tissue and tumor radiosensitivity and to determine the CTCAE severity grade with a high statistical performance [23]. Biomarker analyses predicting anti-angiogenic efficacy and immunostimulatory effects of treatment will be also performed to understand the association of these markers with treatment response (on blood and tumor samples at screening and post-treatment).

### Recruitment

Enrolment started in November 2023 and is currently ongoing with an expected trial duration of 36 months, including treatment and follow-up.

## Discussion

Several studies have demonstrated the utility of PSMA PET imaging for the staging and diagnostic of recurrence in patient with ccRCC. Up to date, only one case has been described relating to the use of PSMA radioligand therapy (PRLT) in RCC [24]. The feasibility of the project PRadR relies on the complementarity of the teams involved in this project: expertise and experience of nuclear medicine departments in managing PRLT in trials and routine activity with a compassionate use; experience of investigators and participating sites selected for this project who will ensure acceptable accrual rate and adequate patient management; expertise of teams involved in managing of the exploratory study. The participating sites are referent institutions in oncology with an important activity in the field of kidney cancer, drawing patients regionally. This selection should ensure a fast recruitment in the expected timeline. In this context, the expected accrual rate of less 1 patient / month / center (45 patients / 24 months / 4 centers) seems clearly reachable.

PRadR study is an exclusively academic project with no participation from industrial partners. Our propose prospective clinical trial, based on solid biological and clinical data, may lead to new indications for the use of ^177^Lu-PSMA-1 in mccRCC patients and should confirm the efficacy of this radionuclide therapy with limited adverse events. PRadR is the first multicenter, phase I-II trial aiming to prospectively define the safety and activity of ^177^Lu-PSMA-1 in mccRCC patients. The ancillary study regarding the dosimetry study would be of great importance. Dosimetry studies have underlined the dose received by the kidneys can vary between patients with a standard deviation as large as 50%. Kidney absorbed dose appeared to differ between PSMA ligands. Indeed, a ~ 1.5x higher median kidney absorbed dose for ^177^Lu-PSMA-1 compared to ^177^Lu-PSMA-617 has been reported without difference in the rate of kidney toxicity [25, 26]. Considering the significant inter-patient variability, the adoption of individualized renal dosimetry is recommended as a standard practice in routine clinical settings for radionuclide therapy. This approach facilitates the customization of therapeutic doses, allowing the delivery of larger doses while minimizing the risk of nephrotoxicity. The estimation of the absorbed dose in several OAR (Organs At Risk) and identifiable lesions will be performed from SPECT images with the dosimetry workflow that has already been developed and used on previous treatments in our institution (Lutathera for neuroendocrine tumors, ^177^Lu-PSMA-1 for prostate cancer) [27–29]. At the end of the study, we will dispose of an individualized estimation of the absorbed dose in all OAR and lesions.

### Electronic supplementary material

Below is the link to the electronic supplementary material.

Supplementary Material 1: PRadR study flow-chart

## Data Availability

The database will be hosted by the Leon Berard Center, Lyon, France. The datasets generated during and/or analyzed during the current study are available from the corresponding author on reasonable request.

## Abbreviations

AE: Adverse Event
ANC: Absolute neutrophil count
ANSM: Agence Nationale de Sécurité du Médicament et des produits de santé
BORR: best overall response rate
CKD-EPI: Chronic Kidney Disease Epidemiology Collaboration
CNS: Central nervous system
CR: Complete response
CT: Computed tomography
CTCAE: Common Terminology Criteria for Adverse Events
DCR24w: Disease Control Rate after 24 weeks of treatment
DoR: Duration of response
IMPD: Investigational Medicinal Product Dossier
IrAE: Immune related Adverse Event
mccRCC: Metastatic Clear-Cell Renal Cell Carcinoma
OAR: Organs At Risk
OR: Objective Reponse
ORR: Objective Response Rate
ORR24w: Objective Response Rate after 24 weeks of treatment
OS: Overall survival
PFS: Progression-free survival
PSMA: Prostate Specific Membrane Antigen
PR: Partial response
RCC: Renal Cell Carcinoma
RECIST: Response Evaluation Criteria In Solid Tumors
SAE: Serious Adverse Event
SD: Stable disease
ST: Incidence of Severe Toxicities
SUSAR: Suspected Unexpected Serious Adverse Reaction
ULN: Upper Limit Of Normal
