# Supplementary material for: A multicentric, single arm, open-label, phase I/II study evaluating PSMA targeted radionuclide therapy in adult patients with metastatic clear cell renal cancer (PRadR)
Source: BMC Cancer. 2024 Feb 1;24:163. doi: 10.1186/s12885-023-11702-8 (PMC10835868; doi:10.1186/s12885-023-11702-8)
Supplement: Supplementary file 1 — Supplementary Material 1: PRadR study flow-chart [file 12885_2023_11702_MOESM1_ESM.docx]

| **PERIOD**  **Week** | **SCREENING PERIOD** | | | | **TREATMENT PERIOD (6-weeks cycle period)** | | | | | | | | | **FOLLOW-UP PERIOD** | | |
| --- | --- | --- | --- | --- | --- | --- | --- | --- | --- | --- | --- | --- | --- | --- | --- | --- |
|  |  |  |  |  | C1 | | | C2 | | C3 | | C4 | | **STSV at W24 or before initiation of new anti-cancer treatment in case of early discontinuation** | **Every 12 weeks until M12** **until** PD, death, withdrawal of consent, initiation of new anti-cancer treatment or overall study completion | **Every 24 weeks**  after M12 and until death, withdrawal of consent, initiation of new anti-cancer treatment or overall study completion |
| **Day** | -42  to -14 | -14 to  -1 | | -7 to  -1 | D1 | D4-D7 | D22 | D1 | D22 | D1 | D22 | D1 | D22 |  |  |  |
| *Window* |  | | | |  | | | | | | | | | *± 1 week* | *± 2 weeks* | *± 2 weeks* |
| ICF signature | X |  | |  |  | | |  | |  | |  | |  |  |  |
| Validation of eligibility criteria | X | | | |  | | |  | |  | |  | |  |  |  |
| Cancer History and Relevant Medical History | X |  | |  |  | | |  | |  | |  | |  |  |  |
| ^68^Ga-PSMA-PET* | X |  | |  |  | | |  | |  | |  | |  |  |  |
| **STUDY TREATMENT (***± 1 week)* | | | | | | | | | | | | | | | | |
| **^177^Lu-PSMA-1 injection (IV)** |  | | | | **X** |  |  | **X** |  | **X** |  | **X** |  |  |  |  |
| **MEDICAL EXAMS** | | | | | | | | | | | | | | | | |
| Physical examination |  | | X |  | X^a^ |  | X | X^a^ | X | X^a^ | X | X^a^ | X | X | X |  |
| PS ECOG |  | | X |  | X^a^ |  |  | X^a^ |  | X^a^ |  | X^a^ |  | X | X |  |
| Vital signs (blood pressure, pulse, temperature, respiratory rate) |  | | X |  | X^b,c^ |  |  | X^b^ |  | X^b^ |  | X^b^ |  | X |  |  |
| ECG |  | | X |  | If clinically indicated | | | | | | | | | If clinically indicated |  |  |
| Hematology |  | |  | X |  |  | X^c^ | X^c^ | X^c^ | X^c^ | X^c^ | X^c^ | X^c^ | X |  |  |
| Biochemistry |  | |  | X |  |  | X^c^ | X^c^ | X^c^ | X^c^ | X^c^ | X^c^ | X^c^ | X |  |  |
| Urinalysis (dipstick, proteinuria if applicable) |  | |  | X |  |  |  | X^c^ |  | X^c^ |  | X^c^ |  | X |  |  |
| Pregnancy test if applicable |  | |  | X |  |  | X | X^a^ | X | X^a^ | X | X^a^ | X | X |  |  |
| HR-QOLs |  | |  |  | X^a^ |  |  |  |  | X^a^ |  |  |  | X | X until PD | X until PD |
| AEs and Concomitant medications | X : SAE related to study procedures | | | | X continuously | | | | | | | | | | X related SAEs | |
| **TUMOR AND SURVIVAL** | | | | | | | | | | | | | | | | |
| Tumor assessment (CT-Scan) | X |  | |  | Between W9 and W10 | | | | | | | | | X if PD not documented before | Q12W up to 1 year thereafter every Q24W if PD not documented before | |
| Brain MRI | X |  | |  | If clinically indicated^d^ | | | | | | | | | |  |  |
| Survival status |  | | | |  | | | | | | | | |  | X | X |

**Keys:** *: last eligibility criteria to be checked **a**: Pre-dose. **b**: Pre-dose and within 60 min post-dose**. c**: within 72 hours. **d:** For all patients, brain MRI is required at baseline to assess the presence of brain metastases and during the study as per investigaror discretion. However, patients with an history of brain metastases must be followed by brain MRI during the study.
